# Glibenclamide-Induced Autophagy Inhibits Its Insulin Secretion-Improving Function in *β* Cells

**DOI:** 10.1155/2019/1265175

**Published:** 2019-08-15

**Authors:** Jiali Zhou, Xincong Kang, Yushuang Luo, Yuju Yuan, Yanyang Wu, Meijun Wang, Dongbo Liu

**Affiliations:** ^1^Horticulture and Landscape College, Hunan Agricultural University, Changsha 410128, China; ^2^State Key Laboratory of Subhealth Intervention Technology, Changsha 410128, China; ^3^College of Food Science and Technology, Hunan Agricultural University, Changsha 410128, China; ^4^Hunan Provincial Key Laboratory of Crop Germplasm Innovation and Utilization, Hunan Agricultural University, Changsha 410128, China; ^5^Hunan Co-Innovation Center for Utilization of Botanical Functional Ingredients, Changsha 410128, China

## Abstract

Diabetes is a metabolic disease, partly due to hypoinsulinism, which affects ∼8% of the world's adult population. Glibenclamide is known to promote insulin secretion by targeting *β* cells. Autophagy as a self-protective mechanism of cells has been widely studied and has particular physiological effects in different tissues or cells. However, the interaction between autophagy and glibenclamide is unclear. In this study, we investigated the role of autophagy in glibenclamide-induced insulin secretion in pancreatic *β* cells. Herein, we showed that glibenclamide promoted insulin release and further activated autophagy through the adenosine 5′-monophosphate (AMP) activated protein kinase (AMPK) pathway in MIN-6 cells. Inhibition of autophagy with autophagy inhibitor 3-methyladenine (3-MA) potentiated the secretory function of glibenclamide further. These results suggest that glibenclamide-induced autophagy plays an inhibitory role in promoting insulin secretion by activating the AMPK pathway instead of altering the mammalian target of rapamycin (mTOR).

## 1. Introduction

Diabetes mellitus (DM) is a metabolic disorder characterized by persistent hyperglycemia. An abnormal increase in blood glucose may be due to a defect in insulin self-secretion caused by the immune system (type 1 diabetes mellitus, T1DM), or resistance to the cellular effects of insulin, as well as insufficient insulin secretion (type 2 diabetes mellitus, T2DM) [1]. According to the International Diabetes Federation (IDF) statistics of 2017, there are 425 million people with diabetes worldwide, and by 2045, that number will reach 629 million, which puts a heavy burden on social and economic development. The drug treatments for diabetes currently include insulin, insulin secretagogues, promotion of peripheral tissue glucose use, inhibition of intestinal glucose absorption, and insulin sensitizers [2].

Sulfonylureas (SUs) are one of the most commonly prescribed classes of drugs for treatment of T2DM [3]. SUs bind to their receptors (sulfonylurea receptor 1, SUR1), which are the regulatory subunits of the ATP-dependent potassium (K_ATP_) channel. Thus, SUs can close the K_ATP_ channel in pancreatic *β* cells, followed by membrane depolarization and open the voltage-dependent Ca^2+^ channels (VDCCs) to increase intracellular calcium (Ca^2+^), resulting in insulin secretion and decreasing blood glucose [4–6]. Glibenclamide is a second-generation SU drug that inhibits SUR1 at nanomolar concentrations and targets K_ATP_ (Sur1-Kir6.2) channels for the treatment of T2DM [7]. Previous studies have shown that glibenclamide can improve insulin secretion at low-normal glucose, which may increase the risk of hypoglycemia when attempting to maintain tight glucose control [8]. *β* cells are the targets of glibenclamide and the only highly differentiated insulin-secreting cell in the human body, which secrete a certain amount of insulin to maintain glucose homeostasis [9].

Autophagy is a self-protective pathway of cell catabolism that allows cells to degrade misfolded proteins or damaged organelles, providing energy for cells and maintaining their homeostasis [10, 11]. Autophagy is either positively mediated by the adenosine 5′-monophosphate (AMP) activated protein kinase (AMPK) pathway [12], or negatively mediated by the mammalian target of rapamycin (mTOR) pathway [13]. Studies have shown that autophagy plays an important role in many organs, especially metabolic organs, which not only maintain the basic homeostasis of cells but also regulate cells' function [14]. Many studies have shown that activation of islet autophagy may enhance antioxidant response, leading to reduced oxidative stress and reduced apoptosis in high glucose stress [15, 16]. Also, vitamin D reduced the incidence of T1DM, enhanced insulin secretion, and relieved pancreatic inflammation in STZ-treated mice via enhancing autophagy in pancreatic *β* cells [10]. However, not all induced autophagy is beneficial. In insulin-producing *β* cells, excess autophagy degrades insulin granules, resulting in decreased insulin contents and systemic glucose intolerance, whereas in insulin-responsive cells, activating autophagy decreases endoplasmic reticulum (ER) stress and improves insulin sensitivity [17, 18]. In short, these reports show that autophagy has different effects and particular physiological functions in different tissues or cells.

Unfortunately, it is not clear whether glibenclamide, an insulin secreting drug, would induce autophagy of *β* cells and have a corresponding effect on its function. In the present study, we showed that glibenclamide can induce autophagy through the AMPK pathway in pancreatic *β* cells. Then, the relationship between autophagy and insulin release was further explored. We conclude that autophagy induced by glibenclamide may inhibit its effect on insulin secretion in *β* cells.

## 2. Materials and Methods

### 2.1. Cell Culture

MIN-6 (provided by Dr. Huang Gan, Xiangya Medical School, China), a mouse pancreatic *β* cell, was cultured in Dulbecco's modified Eagle's medium (DMEM, Biological Industries, Israel, 0012418) containing 25 mM glucose and supplemented with 10% fetal bovine serum (FBS, Biological Industries, Israel, 1822477), 1% 100× L-glutamine (TransGen, China, L71103), and 0.4% *β*-mercaptoethanol (Santa Cruz, USA, SC-202966). Cultures were grown in a high-humidity environment with 5% carbon dioxide (CO_2_) at a temperature of 37°C. Cells were used in experiments after two passages.

### 2.2. Insulin Secretion Assay

The insulin secretion assay was performed as previously described [19]. Briefly, MIN-6 cells were incubated for 30 min in Krebs–Ringer HEPES buffer (130 mM NaCl, 4.7 mM KCl, 0.5 mM NaH_2_PO_4_, 0.5 mM MgCl_2_, 1.5 mM CaCl_2_, and 10 mM HEPES-NaOH (pH 7.4)) supplemented with 0.5% BSA and 2.8 mM glucose (Sigma, USA, G7528). Subsequently, MIN-6 cells were incubated for 4 h in 1640 medium as control, 10 *μ*M glibenclamide [20] (Sigma, USA, G0639), and 10 *μ*M glibenclamide with 2 mM 3-MA. After 4 h, the supernatant was collected and the insulin levels were measured using an ELISA Kit (Millipore, Germany, EZRMI-13K). Data were normalized to nontreated control cells.

### 2.3. Immunofluorescence Staining

Immunofluorescence staining was used to assess autophagosome formation [21]. MIN-6 cells were plated on 10 mm glass coverslips in 24-well plates cultured with DMEM and allowed to grow for 12 h. Then, MIN-6 cells were treated with different conditions. After 4 h of incubation at 37°C, the medium was discarded. The cells were incubated with 4% paraformaldehyde for 10 minutes after being washed three times with phosphate-buffered saline (PBS) and then washed again with PBS three times. After blocking with blocking liquid (PBS with 10% normal goat serum) for 30 min, cells were incubated at 37°C for 1 h with a primary antibody, anti-LC3 (microtubule-associated protein 1 light chain 3) polyclonal antibody (Medical & Biological Laboratories Co., Ltd., Japan, PM036), and washed three times with blocking liquid. Cells were subsequently incubated at 37°C for 1 h with a secondary antibody, anti-rabbit IgG Alexa Fluor 488 conjugate (Cell Signaling Technology, USA, 4412), and then washed three times with PBS. Imaging was performed with a confocal laser scanning microscope (Zeiss LSM710).

### 2.4. Western Blotting

Western blotting is used to assess the expression of proteins with different molecular weights. MIN-6 cells were cultured in 6-well plates and treated as described in the previous section. After washing three times with PBS, cells were lysed in 200 *μ*L of 2% sodium dodecyl sulfate (SDS) per well. The extracts were heated to 100°C for 10 min and then mixed with 6× protein loading buffer (TransGen, China, J21020) and heated again to 100°C for 10 min. The extracts were separated by SDS-PAGE and then transferred to a polyvinylidene difluoride membrane (PVDF). After blocking with 5% nonfat milk in PBST (PBS plus 0.2% Tween-20) for 1 h, the membrane was stained with multiple primary antibodies: anti-LC3 (Medical & Biological Laboratories Co., Ltd., Japan, PM036); anti-S6K (ribosomal protein S6 kinase) antibody (Cell Signaling Technology, USA, 2708); anti-phospho-S6K antibody (Cell Signaling Technology, USA, 9206); anti-mTOR antibody (Cell Signaling Technology, USA, 2983); anti-phospho-mTOR antibody (Cell Signaling Technology, USA, 2974); anti-AMPK antibody (Cell Signaling Technology, USA, 4811); anti-phospho-AMPK antibody (Cell Signaling Technology, USA, 4188); and anti-GAPDH (glyceraldehyde-3-phosphate dehydrogenase) antibody (Yataihengxin, China, ZB002) at 4°C overnight followed by a secondary antibody of goat anti-mouse IgG1 (Southern Biotech, USA, 1070-05) or goat anti-rabbit IgG (Southern Biotech, USA, 4050-05) for 1 h. After washing with PBS, a detection step with ECL western blotting detection reagents (Pierce, USA, 32106) was conducted. The chemiluminescent intensities of protein signals were quantified using Image J 1.47v software (National Institutes of Health, USA).

### 2.5. Statistical Analysis

Data are presented as mean ± SEM, and the standard errors of the mean in the current study were based on triplicate samples. Statistical comparisons were assessed using Student's *t*-tests. *P* < 0.05 was considered a statistically significant difference, and *P* < 0.01 was considered a highly significant difference.

## 3. Results

### 3.1. Glibenclamide Improved Insulin Secretion in MIN-6 Cell Model

**Figure 1:**
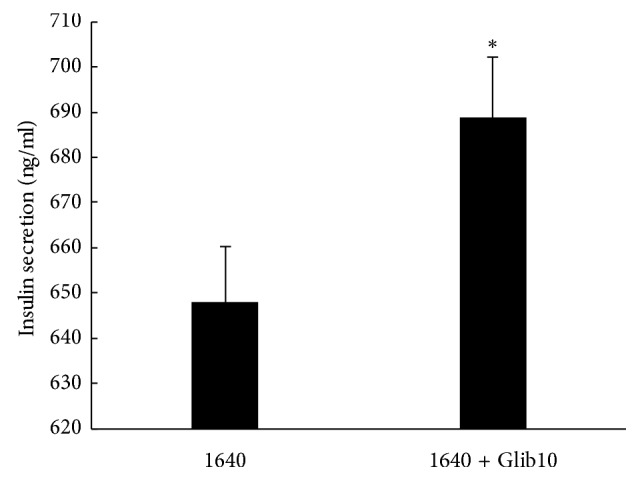
Effect of glibenclamide on insulin secretion in 1640 medium. MIN-6 cells were treated with or without 10 *μ*M glibenclamide in 1640 medium (10 mM glucose) for 4 h. Supernatant was collected, and the insulin level was measured using an ELISA kit. ^*∗*^*P* < 0.05, 1640 vs. 1640 + Glib10. Data shown are mean ± SEM of values from three experiments with triplicate samples.

To demonstrate the effect of glibenclamide on the function of islet cells, MIN-6 cells were cultured in 1640 medium with or without 10 *μ*M glibenclamide for 4 h, then the supernatant was collected, and insulin level was measured using an ELISA kit. The insulin level was significantly increased after treatment with glibenclamide (Figure 1), indicating that glibenclamide can stimulate insulin secretion in a MIN-6 cell model.

### 3.2. Glibenclamide Induced Autophagy of MIN-6 Cells via the AMPK Pathway

**Figure 2:**
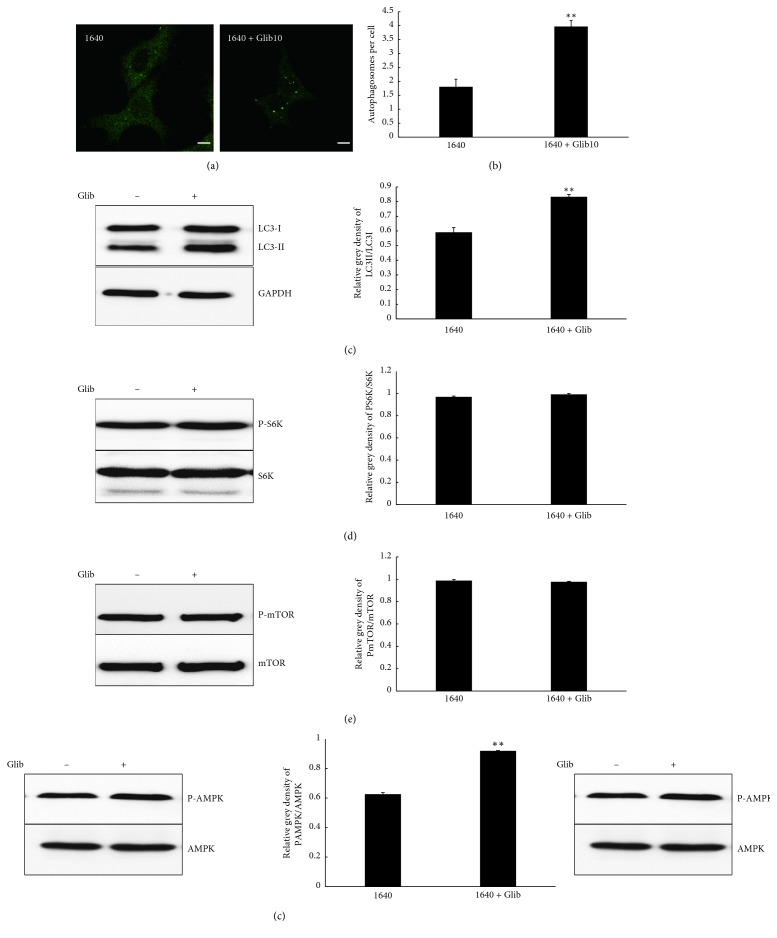
Glibenclamide induced autophagy in MIN-6 cells through the AMPK pathway. (a) Cell immunofluorescence analysis. MIN-6 cells were cultured with or without 10 *μ*M glibenclamide in 1640 medium and stained with an anti-LC3 antibody and then observed using a laser confocal microscope. Scale bar: 5 *μ*m. (b) Statistical results of the cell immunofluorescence test. ^*∗∗*^*P* < 0.01, 1640 vs. 1640 + Glib10. Data shown are mean ± SEM of values from at least 30 cells with triplicate samples. (c–f) Western blot analysis and statistical results of protein quantification. MIN-6 cells were immunoblotted with different antibodies against (c) LC3 and GAPDH; (d) P-S6K and S6K; (e) P-mTOR and m-TOR; and (f) P-AMPK and AMPK.

To investigate the role of glibenclamide in autophagy of MIN-6 cells, we detected LC3, the iconic autophagy protein, and the autophagy pathway. Because LC3-II level is correlated with the number of autophagosomes, the ratios of LC3-II to LC3-I levels in cells are considered an accurate indicator of autophagy activity. After glibenclamide treatment for 4 h, the number of autophagosomes was significantly greater (Figures 2(a) and 2(b)), and the conversion to LC3-II was clearly increased (Figure 2(c)). The protein results confirmed the conclusion of cellular immunofluorescence. The phosphorylation level of S6K, a substrate of mTOR (Figure 2(d)), and total mTOR were unchanged (Figure 2(e)), while the phosphorylation of AMPK was activated (Figure 2(f)). These results suggested that glibenclamide induced autophagy in MIN-6 cells via the AMPK pathway.

### 3.3. Autophagy Inhibited the Effect of Glibenclamide on Insulin Secretion in MIN-6 Cells

**Figure 3:**
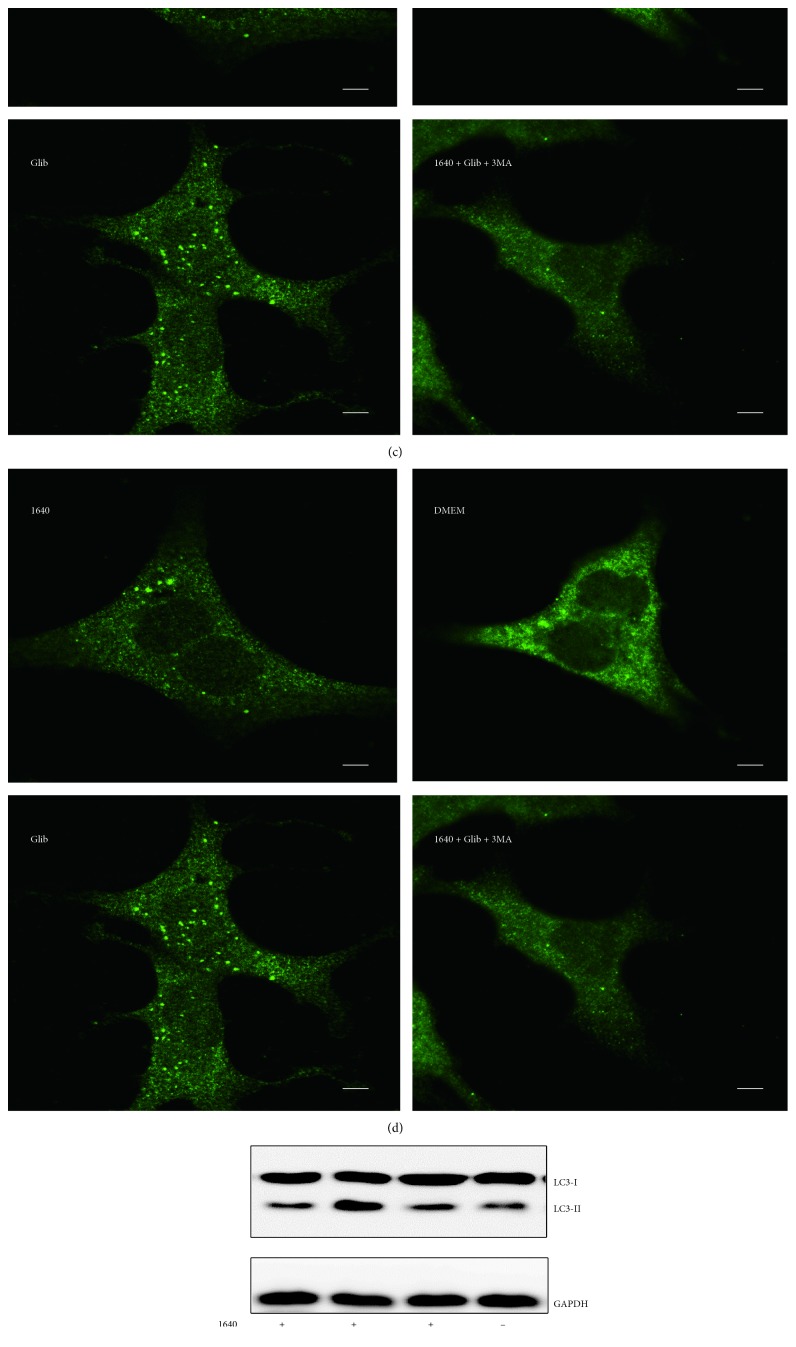
Glibenclamide-induced autophagy may inhibit its ability to promote insulin secretion in MIN-6 cells. (a) The different treatments of MIN-6 cells for insulin secretion level; ^*∗*^*P* < 0.05, 1640 vs. 1640 + Glib10; ^*∗∗*^*P* < 0.01, 1640 vs. 1640 + Glib10 + 3-MA. (b) Statistical results of cell immunofluorescence test. ^*∗*^*P* < 0.05, 1640 vs. 1640 + Glib10. Data shown are mean ± SEM of values from at least 30 cells with triplicate samples. (c) Cell immunofluorescence analysis. MIN-6 cells were stained with an anti-LC3 antibody and observed with a laser confocal microscope (scale bar: 5 *μ*m). (d) LC3 turnover assay and the relative grey density of LC3II/LC3I.

To explore the importance of autophagy in insulin secretion, we investigated whether inhibited autophagy affected insulin release. The insulin level was greatly improved by glibenclamide and increased further when 3-MA (an autophagy inhibitor) was added (Figure 3(a)). Meanwhile, autophagy results were consistent with our previous observation (Figures 3(b)–3(e)). These results demonstrated that glibenclamide-induced autophagy inhibited its insulin promoting function in MIN-6 cells.

## 4. Discussion

In this investigation, glibenclamide showed a significant positive effect on insulin secretion as reported previously [8, 22]. Like other SUs, glibenclamide increases insulin secretion by directly closing ATP-sensitive K^+^ channels in pancreatic *β* cells, causing membrane depolarization, opening voltage-dependent Ca^2+^ channels, and leading to influx and elevation of intracellular Ca^2+^, triggering exocytosis of insulin [23, 24]. Moreover, we found that glibenclamide induced autophagy through the AMPK pathway in MIN-6 cells. To further explore the relationship between autophagy and insulin release, we added autophagy inhibitor 3-MA to inhibit autophagy induced by glibenclamide. When 3-MA was added with glibenclamide, the insulin level was much higher compared with that of glibenclamide alone. Our findings are in agreement with the previous studies showing that inhibiting autophagy enhanced insulin secretion in MIN-6 cells or INS-1E cells [25, 26].

Glibenclamide promotes the secretion of insulin directly without stimulating proinsulin biosynthesis [27]. The mechanism of glibenclamide function is regulating the opening and closing of calcium channels and depends on the vesicular Cl^−^ flux, which initiates the intracellular acidification of insulin secretory granules, to promote the release of insulin particles [28]. Meanwhile, other reports showed that a high level of proinsulin was rapidly transferred to autophagy and directed to lysosomal degradation [26]. The degradation of insulin particles is mainly mediated by microautophagy and endocytosis, while proinsulin degradation is mediated by macroautophagy [26]. Goginashvili et al. also showed that *β* cells induced lysosomal degradation of proinsulin through PKD (protein kinase D) under starvation [29]. When autophagy activates, degradation of proinsulin increases via the endoplasmic reticulum pathway, resulting in decreased insulin secretion. That may be the most definitive reason for our finding that glibenclamide-induced autophagy inhibits its insulin secretion, improving function.

On this basis, it would be interesting to use glibenclamide together with autophagy inhibitors after meals to strengthen the efficacy and buffer the hypoglycemic risk in patients with low-normal glucose. However, specific inhibition of autophagy in *β* cells probably improves the efficacy of SUs, but systemic autophagy knockout may have a negative effect. It was shown that impaired autophagy led to accumulation of human islet-amyloid polypeptide (hIAPP) and exacerbated hIAPP-induced *β* cell toxicity [30, 31]. Studies on *β*-cell-specific atg7 knockout mice also have shown that loss of autophagy reduces glucose tolerance [32]. Hence, further research is required for precise guidance on the combined use of glibenclamide and autophagy inhibitors.

## 5. Conclusions

1. Glibenclamide induced autophagy in MIN-6 cells via the AMPK pathway, which provides a new direction for the study of the mechanism of sulfonylureas.
2. When glibenclamide-induced autophagy was inhibited, the insulin secretion level was enhanced in MIN-6 cells.
3. Sulfonylurea should be used together with autophagy inhibitors after meals to strengthen the efficacy and buffer the hypoglycemic risk in patients with low-normal glucose. Greater emphasis should be placed on the interaction between autophagy and clinical drug.

## Data Availability

The data used to support the findings of this study are available from the corresponding author upon request.
